# Supplementary material for: CD28 and 41BB Costimulatory Domains Alone or in Combination Differentially Influence Cell Surface Dynamics and Organization of Chimeric Antigen Receptors and Early Activation of CAR T Cells
Source: Cancers (Basel). 2023 Jun 7;15(12):3081. doi: 10.3390/cancers15123081 (PMC10295952; doi:10.3390/cancers15123081)
Supplement: Supplementary file 1 [file cancers-15-03081-s001.zip › cancers-2439084 Supplementary Figures.pdf]

Supplementary Materials

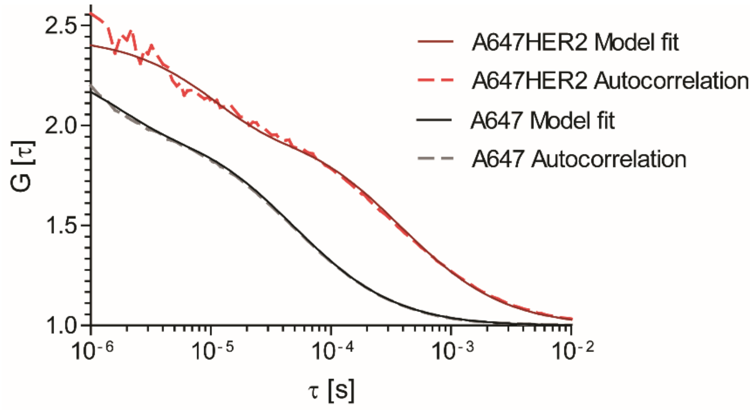

$\tau_{d\ A647}$ [µs]	S [-]	ω_r [µm]	$\tau_{d\ A647HER2}$ [µs]
44.18 ± 5.02	6.80 ± 0.51	0.24 ± 0.01	383.303 ± 4.790

Figure S1. Calibrating the instrumental parameters and measuring the diffusion correlation time of the Alexa Fluor 647 conjugated monomer HER2 ECD for FCS experiments. Representative model fits are shown. Diffusion correlation time τ_d and the instrumental parameter S were determined by fitting a model with free 3D diffusion and triplet state correction for free Alexa Fluor 647 dye. The lateral focus radius ω_r was calculated according to Equation 2. The diffusion correlation time of the Alexa Fluor 647 conjugated monomer HER2 ECD was determined by fitting a model with free 3D diffusion and triplet state correction using the previously calibrated S and ω_r values as constants.

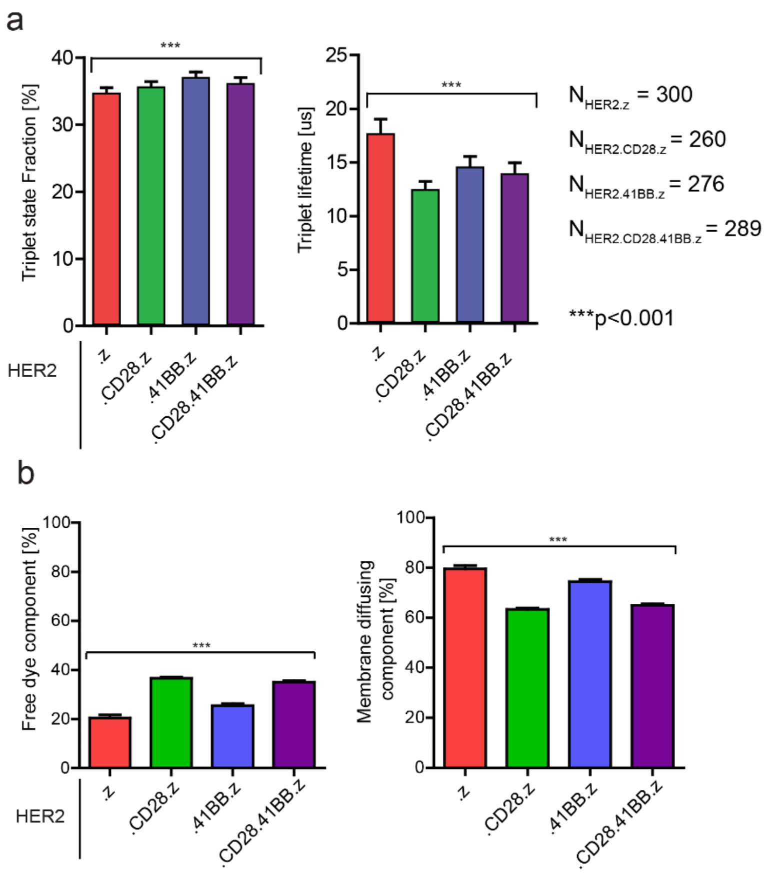

Figure S2. Triplet state fraction, triplet lifetime, and the fractional distribution of the diffusing components in FCS measurements performed on live unstimulated CAR T cells. (a) The triplet state

fraction and lifetime were fitted parameters, while the structural parameter *S* and the diffusion correlation time of the free Alexa Fluor 647 monomer HER2 ECD were fixed at values determined in control experiments. (b) The fractional distribution of Alexa Fluor 647 conjugated monomer HER2 ECD species detached from the cells diffusing freely in 3D and of the CAR species diffusing in 2D in the membrane, tagged by the Alexa Fluor 647 conjugated monomer HER2 ECD. The charts represent mean ± SD; nHER2.z=300, 4 donors; nHER2.CD28.z=260, 3 donors; nHER2.41BB.z=276, 4 donors; nHER2.CD28.41BB.z=289, 4 donors; ***p<0.001.

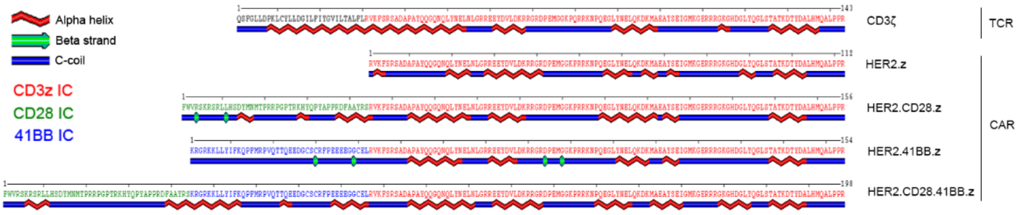

Figure S3. Modeling the tertiary structure of the native CD3ζ and HER2-specific chimeric antigen receptors using the RoseTTAFold deep learning-based protein structure prediction algorithm. The diagram illustrates the secondary structure of the native CD3ζ and the intracellular domains of the HER2.z, HER2.CD28.z, HER2.41BB.z, and HER2.CD28.41BB.z CAR constructs. The CD3z, CD28, and 41BB intracellular costimulatory domains are indicated by the color coding of amino acid names.

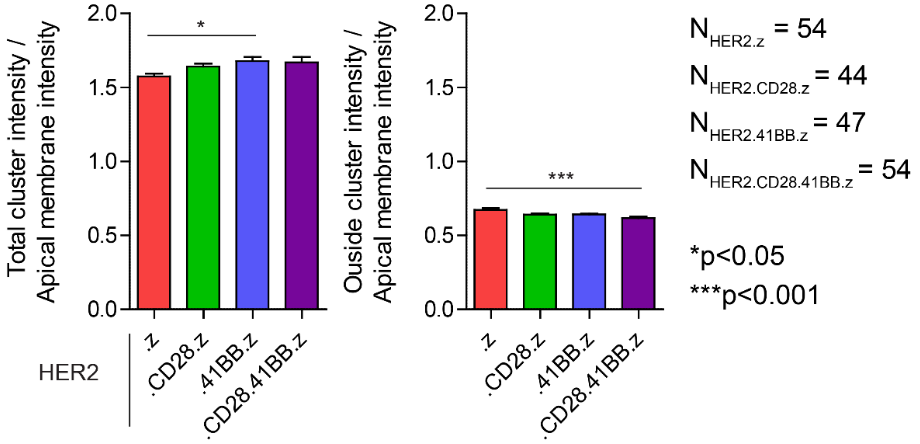

Figure S4. Determining the relative amount of receptors localized in clusters. CAR molecules were labeled with Alexa Fluor 647 conjugated HER2 ECD, and their membrane distribution on the surface of unstimulated T cells was investigated using super-resolution AiryScan microscopy. Approximately 600 nm thin optical slices of the apical membranes were imaged, segmented and analyzed. The charts represent mean ± SEM; nHER2.z=54, 4 donors; nHER2.CD28.z=44, 3 donors; nHER2.41BB.z=47, 4 donors; nHER2.CD28.41BB.z=54, 4 donors; *p<0.05, ***p<0.001.
